# Optimizing Liver Cancer Care Through BCLC Principles

**DOI:** 10.1055/a-2769-7224

**Published:** 2026-02-27

**Authors:** Gemma Iserte, Neus Llarch, Andrew M. Moon, María Reig

**Affiliations:** 1 Liver Oncology Unit, Liver Unit, Hospital Clínic, Barcelona, Spain; 2 Barcelona Clinic Liver Cancer (BCLC) Group, Institut d'Investigacions Biomèdiques August Pi i Sunyer (IDIBAPS), Barcelona, Spain; 3 Centro de Investigación Biomédica en Red de Enfermedades Hepáticas y Digestivas (CIBEREHD), Barcelona, Spain; 4 Department of Medicine, Universitat de Barcelona (UB), Barcelona, Spain; 5 Division of Gastroenterology and Hepatology, University of North Carolina, Chapel Hill, North Carolina, United States; 6 Lineberger Comprehensive Cancer Center, University of North Carolina, Chapel Hill, North Carolina, United States

**Keywords:** shared decision-making, process improvements, quality improvement, nursing, innovation

## Abstract

**Abstract:**

Hepatocellular carcinoma (HCC) care is rapidly evolving, driven by a relentless pursuit of improved patient outcomes, enhanced quality of life, and increased patient empowerment. This transformation draws inspiration from innovative concepts originating in the corporate world, notably the insights of Rita McGrath, and their application to medicine, particularly within the Barcelona Clinic Liver Cancer (BCLC) system. This review explores pivotal advancements in shared decision-making, cross-disease insights, remote monitoring and telehealth, dynamics of multidisciplinary teamwork, and patient empowerment. These innovations signify a fundamental shift in our commitment to alleviating patient distress, fostering treatment adherence, and elevating the overall patient experience. This approach not only optimizes established practices but also broadens the horizons of HCC care by transferring expertise to new domains. Advanced practice nurses (APNs) play an essential role in spearheading this transformation through patient-centered care coordination and educational initiatives, fundamentally improving the management and outcomes of HCC care within the BCLC framework.

**Key Takeaways for Practice and Policy:**

- **Value-based Coordination:** Dynamic scheduling, team coordination, and personalized care reduce inefficiencies, ensuring timely, patient-centered interventions.
- **Empowering Nurses and Patients:** Advanced practice nurses (APNs) play a central role in educating and guiding patients, boosting adherence and autonomy.
- **Leveraging Technology:** Tools like telemedicine and real-time alerts streamline communication, reduce hospitalizations, and optimize resource allocation.
- **Scalable Solutions:** The Barcelona Clinic Liver Cancer (BCLC) model offers replicable practices adaptable to broader healthcare systems.

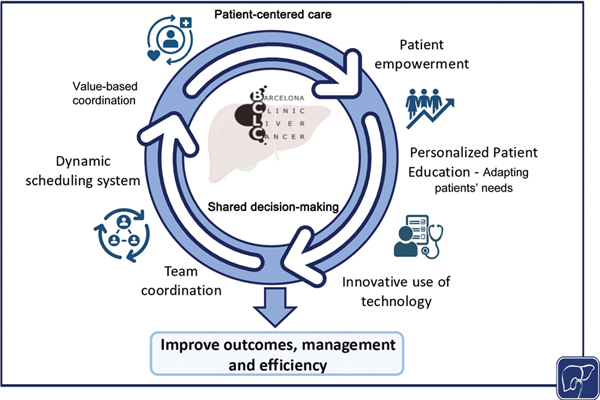

## Introduction

In the ever-evolving landscape of healthcare, innovation plays a pivotal role in driving progress. This holds especially true when considering the complex challenges presented by hepatocellular carcinoma (HCC) and the strategies outlined by the Barcelona Clinic Liver Cancer (BCLC) system. 1 Cutting-edge developments have the potential to revolutionize the approach to HCC care, offering the promise of enhanced patient outcomes and a transformative impact in the field of medicine. There are significant opportunities waiting to be explored within the domain of liver cancer care and the framework provided by BCLC.

HCC care has witnessed a paradigm change that is reshaping the approach to HCC care and enhancing traditional methods with new strategies. Innovations span from optimizing existing practices, such as improving the efficiency of multidisciplinary tumor board approaches, to extending successful methods to new domains, like adapting patient education materials for emerging treatments. 2 3 Transformational innovations, exemplified by the approval of several new systemic treatments over the past 15 years and the recent introduction of immunotherapy combinations, mark a significant turning point in treatment options, survival, and patient care.

The BCLC system stands as a widely acclaimed and recommended algorithm for HCC management. 1 It serves as a standard approach for staging and treatment allocation in HCC and garners endorsement from numerous global scientific and medical associations. 4 5 The BCLC system offers a framework for categorizing HCC patients into five stages based on criteria including tumor size, number, extrahepatic spread, liver function, and overall health. It subsequently proposes tailored treatment strategies and interventions for each HCC stage, spanning from curative intent options like surgery and transplantation for early-stage HCC to locoregional or systemic treatment care for intermediate- or advanced-stage disease.

The most recent BCLC update was praised for its inclusion of a section outlining the intricacies of clinical decision-making to inform a personalized HCC treatment approach for physicians and multidisciplinary tumor boards. 1 This update extends beyond clinical characteristics to encompass the patient's overall health, values, and treatment accessibility. It serves as a guiding beacon for healthcare providers, aiding them in crafting treatment decisions tailored to individual patient needs.

In light of this complexity and need for tailored care, continuous communication and coordination is essential in the care of HCC. In this context, nursing assumes a pivotal role, not only facilitating patient access but also enriching the care experience. Furthermore, an adaptative approach from nurses is key to ensure these tasks and to understand patient's needs. Here we explore the role of innovation, including the evolving role of the nurse, in liver cancer care and the BCLC philosophy.

## BCLC Embracing the Concept of Transient Advantage

Rita McGrath's concept of the transient nature of competitive advantage and the imperative need for continuous adaptation and innovation originates from the dynamic world of business strategy. In *The End of Competitive Advantage: How to Keep Your Strategy Moving as Fast as Your Business* (2013), 6 McGrath proposes that competitive advantages are inherently temporary. In an ever-changing environment, adaptability, learning, and responsiveness are more valuable than static strategic positions. She emphasizes the importance of recognizing when existing advantages are eroding to enable timely reallocation of resources and pursuit of new opportunities. Her ideas resonate with the BCLC philosophy, which similarly requires flexibility and continuous innovation to integrate emerging evidence and technologies into patient care. Indeed, the most recent version of the BCLC strategy incorporates the concept of CUSE (Complexity, Uncertainty, Subjectivity, and Emotion), 1 a framework co-created through patient focus groups and family interviews to better capture the realities of clinical decision-making. CUSE provides a structured lens for translating evidence-based recommendations into context-sensitive care plans. It maintains that evidence and safety remain the starting point of any decision, but recognizes that, in situations of uncertainty, 7 the final choice must also consider other dimensions such as deliverability, patient values, and emotional context. This integration reflects the BCLC group's commitment to continuous improvement through active listening to patients and the transformation of their insights into practical and ethically grounded updates to the BCLC framework.

The initial publication of the BCLC classification in 1999 8 marked a transformative milestone in HCC management. This innovation sparked a paradigm shift within hepatic oncology, underscoring the importance of staying at the forefront of clinical practices and treatment methodologies. Acknowledging the ever-evolving landscape of cancer treatment, including HCC, BCLC recognizes that fixed treatment algorithms or protocols may have diminishing effectiveness over time. As a result, BCLC has remained agile and responsive, continuously adapting its treatment strategies in light of emerging evidence and novel therapies. This evolution necessitates moving beyond rigid frameworks toward an adaptive approach that prioritizes the timely incorporation of new data, multidisciplinary insights, and innovative concepts—such as the clinical decision-making framework introduced in the 2025 BCLC update, and the CUSE concept incorporated in the most recent BCLC classification, 1 which expands the model to integrate patient perspectives and contextual decision-making.

## Patient-Centered Care Coordination

By embracing these principles, the BCLC philosophy adopts a forward-thinking approach that incorporates “shared decision-making” and “value-based medicine” into the decision-making process. 9 The coordination of patients, caregivers, and multidisciplinary clinicians is essential for delivering value-driven healthcare. There are several elements that serve as examples of value-driven healthcare within the BCLC system ( Table 1 ).

**Table 1:** Examples of patient-centered care coordination at BCLC

Practice	Definition	Example
Dynamic scheduling system	Standardized schedule of visits for each treatment adapted to the needs of each patient. The follow-up frequency after specific treatments is protocolized while allowing for unscheduled visits if needed based on medical needs or patient preferences. The dynamic scheduling system is led by the nursing staff.	After 1 week of chemoembolization the advanced practice nurse calls the patient to see how the patient is doing and to assess for symptoms of post-embolization syndrome. During the call, the patient describes a large nodule in femoral area and a low-grade fever. The nurse schedules an extra visit to assess the patient even though the protocolized TACE schedule indicates that the next visit is 6 weeks after treatment.
Team coordination	All professionals involved with patients are in continuous contact to provide the best care for patients and caregivers. Each member knows its role and responsibilities and performs them allowing the workflow of the rest of the team. Continuous communication within the multidisciplinary team to decrease waiting periods.	The multidisciplinary team is formed by all the professionals of the group (hepatologists, radiologists, surgeons, nurses, and other specialists) and each one is aware of their role and tasks, which optimizes patient time and health system resources. Coordinate multiple medical visits on the same day, such as blood tests, CT scans, or appointments with different physicians.
Empowerment of advanced practice nurses	The advanced practice nurse accompanies the patient and caregiver throughout their healthcare process, serving as primary facilitator between the patient and the medical team. In addition, their role is to empower the patients with knowledge and skills in their new situation and/or treatment to make them as autonomous as possible. The knowledge and skills of advanced practice nurses are suited to solve common problems that arise for patients with HCC, which saves time of other professionals and the healthcare resources of the system.	Nurses are empowered to educate patients about the disease and the different treatments available. They develop educational programs that explain what the treatment consists of, dosage, warning signs, possible adverse effects, and how to manage them. Logistic and contact information is also included. All patients receive therapeutic education by the advanced practice nurse in order to empower patients in self-care and their disease process. ( Figs. 1 and 2 are examples of educational program graphic support.)
Asynchronous visits	Asynchronous visits, also known as electronic consults, are useful to be informed about the patient process and be able to decide next steps. In these visits, the professional doesn't have direct interaction with the patient but they have interaction with the patient's medical record or some other professional to talk about the patient situation.	The patient is awaiting results of complementary tests to decide on treatment. An asynchronous visit would allow medical providers to review the result in advance in order to define and organize treatment before the patient's face-to-face visit.
Streamlined coordination process	The committee is organized to ensure that the necessary patient information is previously shared with the entire multidisciplinary team, so that all the professionals involved would have been able to review the case and time is optimized. Each member involved in the committee is assigned a specific task to perform during the week (to prepare the committee) or during the committee to ensure greater efficiency and fluidity.	A patient receives cross-sectional imaging that shows potential progression, which may necessitate a change in treatment. All medical providers review the case 24 to 48 hours prior to tumor board. It is discussed in committee and treatment recommendations are formulated. The APN arranges a call or visit between the patient and treating physician to communicate results 24 to 48 hours after the committee.
Integration of palliative and supportive care	Palliative and supportive care are integrated as structured therapeutic components, ensuring coordinated, patient-centered management that emphasizes continuity of care, optimal symptom control, and comprehensive psychosocial support when disease-directed interventions are no longer appropriate.	In our outpatient clinic, palliative care is initiated when a patient with HCC experiences disease progression or when further oncologic treatment is considered no longer beneficial. Together with the patient and family, care goals are redefined, and the social worker is contacted to coordinate the involvement of the home-based palliative care team.
Innovative use of technology	The use of new technologies helps to provide coordinated, value-based care that has a positive impact on the patient and the caregivers while optimizing resources.	A patient with HCC arrives in the emergency department, generating an electronic alert for the medical team. This allows medical providers to follow up on the patient and his or her progress in a rapid and coordinated manner.

Abbreviations: APN, advanced practice nurse; BCLC, Barcelona Clinic Liver Cancer; HCC, hepatocellular carcinoma.

**Fig. 1:**
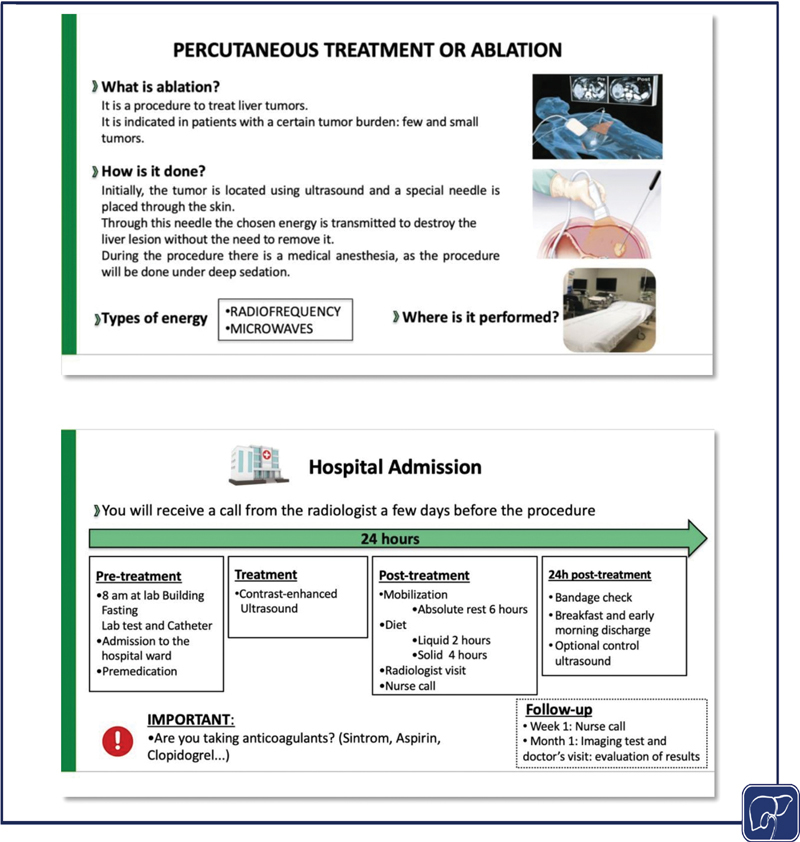
Graphic support for therapeutic education on percutaneous treatment in hepatocellular carcinoma (HCC).

**Fig. 2:**
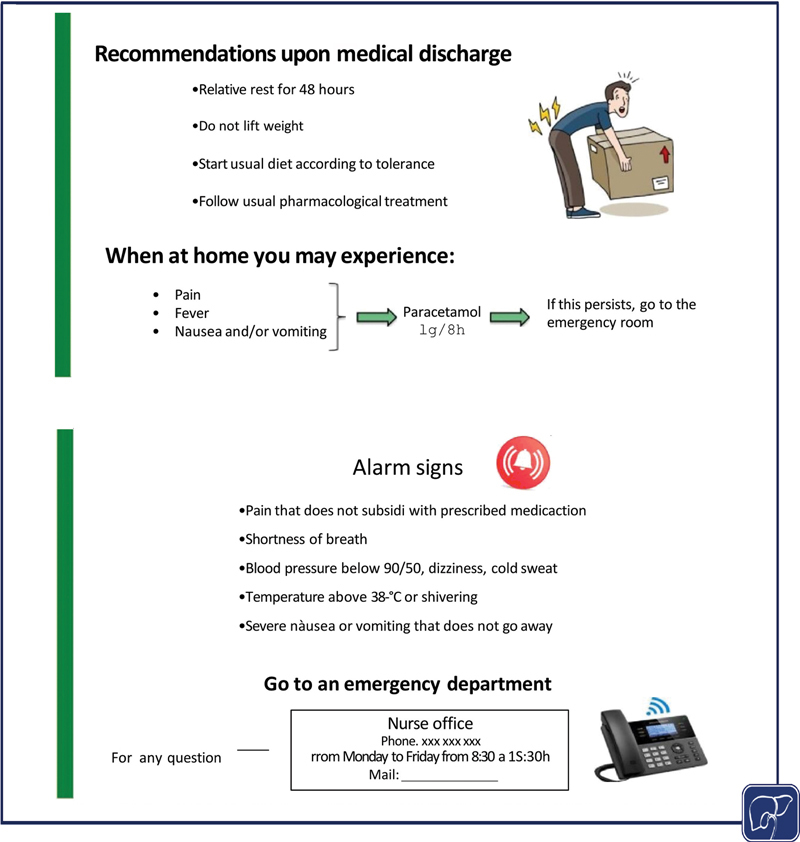
Patient leaflet about immunotherapy recommendations.

### Dynamic Scheduling System

The BCLC group employs a dynamic scheduling system managed by nursing staff. This system ensures flexible and timely access to care for each patient, considering the patient's unique needs and treatment journey, aligning with shared decision-making principles. This system aims to expedite processes, thus minimizing patient wait times throughout their patient journey, while also considering patient preferences.

This workflow commences prior to the patient's initial clinical encounter. Thus, patients can be scheduled quickly, facilitating rapid diagnosis and initiation of treatment without delay. The various follow-up protocols are established in consensus with the multidisciplinary team and are designed by nursing staff. These protocols set forth the schedule for visits and ancillary testing. Flexibility is built into these protocols to incorporate patient preferences and medical needs. This dynamic coordination system allows for the scheduling of as-needed visits based on the patient's needs within a 24- to 48-hour timeframe. Such flexibility diminishes the reliance on emergency services and reduces waiting times.

### Immediate Coordination Between Team Providers

Co-location of multidisciplinary providers (physicians of multiple specialties and advanced practice nurses [APNs]) facilitates rapid communication and decision-making, both tenets of value-based medicine. 9

There is smooth and active communication among the members of the multidisciplinary team through face-to-face communication or digital communication, which facilitates swift decision-making and prompt response to patient needs. Beyond the team members, an understanding of the operations across various departments and sections of the hospital enhances coordination and efficiency.

### Empowerment of Advanced Practice Nurses (APNs)

The BCLC integrated the APN as a core member of the multidisciplinary team, recognizing the breadth, autonomy, and clinical impact of this professional role. Each APN holds a master's degree and possesses the specialized expertise and advanced competencies required for complex clinical decision-making. Their scope of practice encompasses direct clinical care, patient education, research, and leadership, consistent with the definition established by the International Council of Nurses (ICN). 10

APNs serve as pivotal coordinators within the BCLC model, fully aligned with the principles of patient-centered and shared decision-making. They have dedicated consultation areas and personalized scheduling systems, enabling them to provide individualized, continuous, and holistic care throughout the patient journey. Their role extends beyond coordination to include proactive patient education, psychosocial support, and advocacy, ensuring that patients are empowered to make informed choices about their treatment. The nursing consultation process is open and dynamic. Patients or caregivers can contact the nursing staff whenever they need to, without the requirement for a prior appointment. This approach reduces anxiety and enhances the quality of care. APNs also serve as patient educators and advocates, helping to support informed decision-making.

### Asynchronous Visits

Implementing asynchronous visits, defined as an electronic consult that can occur without the need for a separate in-person patient visit or any contact with the patient, represents an innovative strategy that optimizes hospital resources and emphasizes efficiency, a core aspect of value-based medicine. This approach enables efficient patient management without the constraints of traditional scheduling, ensuring timely care. This allows physicians and nurses to revisit treatment strategies and consult clinicians of other specialties in between patients' in-person appointments. This ensures that treatment plans are modified before a patient's visit, thus improving efficiency of care.

### Committee-Streamlined Coordination Process

The BCLC group has developed a streamlined process for managing patients presented in committees, including multidisciplinary tumor boards. This process has expanded beyond the traditional multidisciplinary tumor board concept, allowing for efficient coordination of treatments, admissions, and follow-ups.

A list of patients and their summaries is distributed 48 to 72 hours before the weekly committee. The radiologists review scans for all patients and the pathologists review biopsy samples prior to the meeting. Physicians outside the BCLC committee who are participating in the patient's care will be invited to the meeting on an ad hoc basis. After a clinical decision is made as part of the committee, APNs will contact patients and coordinate care. Each committee has a report which is part of the BCLC archive, which is included in the patient's electronic medical record.

### Integration of Palliative and Supportive Care

Within the BCLC multidisciplinary framework, palliative and best supportive care are regarded as structured and coordinated therapeutic options, rather than as separate or subsequent stages of management. When disease-directed therapies are no longer appropriate or aligned with patient goals, palliative care is discussed within the multidisciplinary committee and incorporated into the individualized care plan. These patients are supported by a dedicated social worker and the hospital's home-based palliative care team, ensuring continuity of care, effective symptom management, and psychosocial support for patients and caregivers.

### Innovative Use of Technology

The use of the “Gomet” alarm system exemplifies a commitment to continuous monitoring and value-driven care coordination, as advocated by value-based medicine. 9 The *Gomet* alarm system is an internal digital tool developed at the Hospital Clínic de Barcelona to improve real-time coordination and patient safety within the transversal Unit model. It acts as a digital clinical footprint, which is activated during the patient's first visit, and automatically generates a clinical alert whenever a liver cancer unit BCLC patient visits the emergency department or is hospitalized.

Each alert is reviewed daily by a designated BCLC team member, who compiles a report and circulates it to the entire multidisciplinary group. This notification process enables the real-time tracking of each patient's clinical trajectory within the institutional information system, ensuring that no episode of care occurs without the team's awareness. It also facilitates seamless communication between inpatient and outpatient teams, allowing for coordinated follow-up and continuity of care immediately after discharge.

This digital tool, integrated into the hospital's electronic health record (EHR) environment, fully complies with institutional and national data protection policies. It represents a practical example of how digital innovation can strengthen multidisciplinary coordination, reduce response times, and enhance patient safety.

## Personalized Patient Education

Personalized education is a key aspect of effective patient management in liver cancer care. Understanding the patient's needs begins with a comprehensive assessment of the individual's medical condition, cultural background, social situation, literacy level, and personal preferences. Tailored educational materials are developed to cater to diverse stages and types of HCC treatments and are continually updated to encompass emerging therapies. Interactive educational sessions encourage patients and caregivers to seek clarification and voice concerns, aiming to improve their understanding of the disease and treatment plan. The incorporation of digital tools for education, such as web-based resources, video tutorials, and mobile applications, enhances accessibility to information. Elicitation of feedback from patients and caregivers and adaptation of educational resources ensure they remain patient- centered. Empowering patients through knowledge equips them to make informed decisions about their care, actively participate in their treatment, and effectively manage their health.

## Incorporation of New Technologies and Formats in Educational Tools

The integration of cutting-edge technologies and innovative formats into educational tools are pivotal in our patient-centered care approach. Digital platforms and online resources provide accessible and up-to-date information to patients. Multimedia and visual aids, such as videos, animations, and infographics, enhance patient comprehension and retention of information.

Data from the BCLC demonstrate the value of these materials. We performed focus groups and interviews to analyze the experience of patients and caregivers, demonstrating that 27% of patients and caregivers refer to information provided during clinic visits and often state that they relied on this information over internet searches. 11

In addition to innovative formats, the BCLC makes use of technology to deliver care virtually. Telehealth and virtual consultations save time and reduce the need for in-person visits. However, tradeoffs regarding patient engagement and an accurate assessment of patient retention of information have to be considered for virtual visits. Customizable and interactive content, including quizzes and personalized treatment trackers, encourage patients to take an active role in managing their health. Continuous updates and incorporation of patient feedback ensure that these tools meet evolving patient needs. Training and empowerment of healthcare professionals is mandatory to ensure they are well-equipped to guide patients in using these resources effectively.

## Managing the Complexity of HCC Care

As we anticipate the future of liver cancer care, the healthcare landscape is poised for profound transformative innovations. These advances span from the expanded integration of telemedicine into HCC management to the exploration of personalized genomics-driven treatments tailored to each patient. These emerging prospects hold tremendous potential for those prepared to pioneer new frontiers in liver cancer care.

The convergence of value-based medicine 9 and translational science within the realm of HCC signifies a significant milestone in the quest for precision medicine. HCC presents distinctive challenges owing to the presence of both cancer and underlying liver disease among HCC patients and the need for coordination among a multidisciplinary care team. Consequently, the integration of value-based medicine and translational science helps patients gain access to therapies rooted in the best available scientific evidence and customized to their unique requirements.

In this new paradigm, HCC care extends beyond the medical sphere alone. Instead, it evolves into a more holistic approach, where patient and caregiver teams play a crucial role. These teams are diverse in their composition, encompassing a rich variety of perspectives and experiences. This is where the leadership of complexity managers comes into play—individuals capable of understanding and navigating the intricate networks of relationships and social dynamics surrounding HCC.

Managing these multifaceted teams involves creating spaces for collaboration, where patients, caregivers, healthcare professionals, and other stakeholders can share knowledge, emotional support, and resources. The diversity of these teams brings a wealth of information and experiences that enrich the understanding of HCC and decision-making in treatment. This innovative approach not only engages the traditional stakeholders but also integrates community leaders, patient advocates, and liver cancer experts. Together, they collaborate in the creation of solutions crafted to the unique needs of each patient and team.

In the end, the management of multifaceted teams heralds a paradigm shift in HCC care. It beckons forth open and collaborative dialogues, where the voices of all involved are not only valued but also harnessed. This represents the frontier of HCC care, one that holds the promise of not only enhancing the quality of life for patients but also driving substantial advancements in the understanding and treatment of this complex disease.

## Concluding Remarks

Rita McGrath's insights into the dynamic nature of competitive advantage have found a meaningful place within the BCLC philosophy. These ideas emphasize the need to stay at the forefront of clinical practices and treatment methodologies, ensuring the organization remains agile and ready to pivot when necessary.

In summary, the amalgamation of innovative insights, the BCLC system's adaptation, and the dedication to patient-centered care coordination propel HCC care into a new era. These groundbreaking concepts are revolutionizing the approach to HCC and are setting new benchmarks for oncology care as a whole.

## Notes

Lay Summary This article explores innovative strategies to enhance liver cancer care by integrating medical and business approaches, emphasizing patient empowerment, teamwork, and advanced technologies to improve outcomes and efficiency.

## References

[1] Reig M Sanduzzi-Zamparelli M Forner A BCLC strategy for prognosis prediction and treatment recommendations: the 2025 update J Hepatol 2025 S0168 8278(25)02571-1 10.1016/j.jhep.2025.10.020 41151697

[2] Salgia R Mendiratta V The multidisciplinary management of hepatocellular carcinoma Clin Liver Dis (Hoboken) 2021 17 06 405 408 34386204 10.1002/cld.1068 PMC8340356

[3] Li J ZH McLeod J Ingledew P A Quality analysis of online patient resources for hepatocellular carcinoma J Clin Gastroenterol 2022 56 01 64 71 33337639 10.1097/MCG.0000000000001477

[4] European Association for the Study of the Liver Sangro B Argemi J Ronot M EASL Clinical Practice Guidelines on the management of hepatocellular carcinoma J Hepatol 2025 82 02 315 374 39690085 10.1016/j.jhep.2024.08.028

[5] Amit Singal C G Llovet J M Yarchoan MAASLD Practice Guidance on Prevention, Diagnosis, and Treatment of Hepatocellular; 2023 10.1097/HEP.0000000000000466 PMC10663390 37199193

[6] Rita Gunther McGrath The End of Competitive Advantage: How to Keep Your Strategy Moving as Fast as Your Business Boston, Massachusetts (MA) Harvard Business Review Press 2013

[7] Ilgen J S Dhaliwal G Educational strategies to prepare trainees for clinical uncertainty N Engl J Med 2025 393 16 1624 1632 41124633 10.1056/NEJMra2408797

[8] Llovet J M Brú C Bruix J Prognosis of hepatocellular carcinoma: the BCLC staging classification Semin Liver Dis 1999 19 03 329 338 10518312 10.1055/s-2007-1007122

[9] Porter M E What is value in health care? N Engl J Med 2010 363 26 2477 2481 21142528 10.1056/NEJMp1011024

[10] Wheeler K J Miller M Pulcini J Gray D Ladd E Rayens M K Advanced practice nursing roles, regulation, education, and practice: a global study Ann Glob Health 2022 88 01 42 35755314 10.5334/aogh.3698 PMC9205376

[11] Iserte G Palou E Llarch N Implication of patients experience in the liver cancer multidisciplinary approach J Hepatol 2023 78 S497 S498

